# Associations between body size and visual impairment of first-year university students in Chongqing: A cross-sectional study

**DOI:** 10.1097/MD.0000000000035763

**Published:** 2024-01-12

**Authors:** Jing Zhang, Ruili Li, Yong Zhang, Cuihong Li, Bingwu Xu, Xiaoya Qi

**Affiliations:** a Department of Health Management, The Second Hospital Affiliated to Chongqing Medical University, Chongqing, China; b School of Public Health, Chongqing Medical University, Chongqing, China.

**Keywords:** body size, college students, first-year students, visual impairment

## Abstract

The relationship between body size and visual impairment (VI) presents a controversial topic in the health sciences. This study aims to evaluate and clarify the potential associations between these 2 variables. We conducted a cross-sectional study on first-year students enrolled in 2022 at the Southwest University of Political Science & Law. The students underwent a series of physical examinations and visual acuity tests. Visual impairment was classified into 3 categories: mild, moderate, or severe. We used logistic regression analysis to examine the association between body size and VI. Our findings indicated a high prevalence of VI among first-year university students; more than 80% of them were affected. In bivariate analysis, height and weight were negatively related to the presence of VI. However, BMI (body mass index) was not related to VI. By adjusting all available confounders, no associations between BMI (OR = 1.002, 95% CI = 0.974–1.032, *P* = .877), height (OR = 0.998, 95% CI = 0.967–1.010, *P* = .298), weight (OR = 0.999, 95% CI = 989–1.009, *P* = .860), and mild-severe VI were found in females. For males, the ORs were 0.988 (95% CI = 0.955–1.021, *P* = .459), 0.980 (95% CI = 0.954–1.006, *P* = .135), and 0.995 (95% CI = 0.985–1.004, *P* = .285) for BMI, height, and weight, respectively. Among young adults demonstrating high academic performance in high schools, the cessation of physical growth, combined with potential eye strain resulting from overuse, may mitigate any previously observed positive associations between physical status and VI in younger children.

## 1. Introduction

Globally, an estimated 100 million people suffer from visual impairment (VI), with prevalence rates alarmingly higher among younger demographics.^[1,2]^ The World Health Organization has identified VI as a priority area under Vision 2020, particularly focusing on school-aged students in developing countries.^[3]^ Various studies have been conducted to identify potential risk factors for VI, including lifestyle changes, near-sighted work, insufficient outdoor activities, academic pressure, and a family history of high myopia.^[4,5]^

Body size has previously been linked to VI, as taller and overweight individuals may have larger eyes and longer axial lengths (AL), potentially leading to myopia-associated VI.^[6]^ For instance, a study conducted in Central China on students aged 6 to 21 found a positive association between overweight or obesity and VI after adjusting for age and sex.^[6]^

However, the relationship between VI and body stature remains inconsistent across studies.^[7,8]^ In South Korea, a survey of individuals aged 5 to 18 revealed that those in higher quintiles of height and weight showed a higher prevalence of myopia.^[9]^ Similarly, taller Chinese children in Singapore typically had lower vision and longer axial lengths.^[10]^ In contrast, taller Chinese adults (aged 41–60) in Singapore had eyes with longer axial lengths without a high prevalence of VI.^[7]^ Interestingly, most previous studies found the correlation between body size and VI prevalence only in children and adolescents, not in adults.^[9]^

Then, we predicted that once body size reached a mature level, the impact of physical state on myopia and VI during growth may diminish. However, the exact age at which this association between body size and VI vanished remained unclear. In this study, we aim to investigate whether the relationship between body size and VI in a young adult with high academic performance still exists.

## 2. Subjects and methods

This study was conducted with a cross-sectional design. All 2022 first-year students at Southwest University of Political Science & Law, a prestigious institution in China where only outstanding students across China have chances to be enrolled, were invited. From them, 201 students who had myopia correction surgery were excluded.

Ethical approval (No. CAF52704054B) was obtained from the Ethics Committee of Chongqing Medical University, according to the principles of the Declaration of Helsinki. Written informed consent was obtained directly from participants. Informed consent was obtained from all subjects involved.

### 2.1. Visual acuity measurement

Two qualified ophthalmologists measured visual acuity (VA) as uncorrected visual acuity (logMAR chart) at a distance of 5 meters. VI considered an uncorrected VA of 20/40 (or less than 20/40) in either eye. VI was classified as mild (between 20/40 and 20/70), moderate (between 20/70 and 20/200), and severe (between 20/200 and 20/400) visual impairment.^[11]^

Because the right eye and the left eye are highly correlated in visual acuity, only the VA of the right eye was used in the analysis as reported before.^[12]^

### 2.2. Physical examination and body mass index definition

Weight (kilograms) and height (centimeters) were measured on a human body scale. body mass index (BMI) was calculated by dividing weight by height (kg/m^2^) and categorized into underweight, normal, overweight, and obese with < 18.5 kg/m^2^, <24 kg/m^2^, <28 kg/m^2^, and ≥ 28 kg/m^2^ cutoffs; respectively.^[13]^

### 2.3. Questionnaire survey

Demographic, sociological, and behavioral information related to eyesight was collected by a self-administrated, web-based questionnaire at the site. The survey was conducted by 8 groups of researchers, including 2 qualified ophthalmologists, 3 ocular assistants, 2 enumerators, and a professional statistical analyst.

### 2.4. Statistical analyses

Two ophthalmologists checked the data, independently. Continuous variables were compared with ANOVA, and the Chi-square test or Fisher’s exact was used to compare categorical variables. The relation between VI and BMI was evaluated with a multiple logistic regression model. *P* < .05 was considered statistically significant. Analyses were performed using the statistical analysis software SPSS 16.0.

## 3. Results

### 3.1. General characteristics of the study participants

Of the 3267 eligible students, 36 participants were excluded due to missing data, and finally, a total of 3231 students (98.9%) were included in the analysis. The characteristics of students aged 16 to 19 are shown in Table 1. Female students with VI in the right eye accounted for 84.6% (1895/2239), among them, mild vision impairment accounted for 11.7% (266/2239), moderate vision impairment accounted for 50.9% (1140/2239), and severe vision impairment accounted for 22.0% (493/2239), only 15.4% (569/3231) was emmetropia. For male students, the prevalence of vision impairment was a little smaller that of females in each group.

**Table 1:** Characteristics of study subjects.

Variable	Female (n = 2239)	Male (n = 992)	*P* value*
Height (cm)	163.1 ± 5.5	175.4 ± 6.2	<.001
Weight (kg)	55.6 ± 11.4	70.1 ± 16.0	<.001
BMI (kg/m^2^)	20.9 ± 4.2	22.7 ± 4.8	<.001
Visual acuity			
logMAR-R	0.79 ± 0.44	0.69 ± 0.46	<.001
logMAR-L	0.74 ± 0.45	0.65 ± 0.47	<.001
Physical status type			
Underweight	562 (25.1)	138 (13.9)	<.001
Normal	1392 (62.2)	548 (55.5)	
Overweight	182 (8.1)	194 (19.6)	
Obese	103 (4.6)	112 (11.3)	
Vision impairment (the right eye)			
No vision impairment	344 (15.4)	225 (22.7)	<.001
Mild vision impairment	262 (11.7)	115 (11.6)	
Moderate vision impairment	1140 (50.9)	464 (46.8)	
Severe vision impairment	493 (22.0)	188 (19.0)	
Parental history of myopia			
Father	720 (32.2)	305 (30.7)	.427
Mother	703 (31.4)	310 (31.3)	.933
Time of owning smartphone			
Primary school	717 (32.0)	231 (23.3)	<.001
Junior middle school	968 (43.2)	410 (41.3)	
Senior high school	321 (14.3)	222 (22.4)	
After college entrance examination	233 (10.4)	129 (13.0)	
Time of boarding at school			
Never	637 (28.5)	294 (29.6)	.099
Junior middle school	993 (44.4)	401 (40.4)	
Senior high school	609 (27.2)	297 (29.9)	
Life caregivers			
Parents	1850 (82.6)	813 (82.0)	.458
Paternal grandparents	252 (11.3)	106 (10.7)	
Maternal grandparents	103 (4.6)	51 (5.1)	
Others	34 (1.5)	22 (2.2)	
Screens used the most of time			
Phone	2108 (94.1)	870 (87.7)	<.001
Panel computer	39 (1.7)	8 (0.8)	
Computer	32 (1.4)	101 (10.2)	
Television	60 (2.7)	13 (1.3)	
Like doing exercises			
Like	856 (38.2)	617 (62.2)	<.001
Dislike	355 (15.6)	68 (6.9)	
In between	1028 (45.9)	307 (30.9)	
Doing exercises			
Occasion	1429 (63.8)	401 (40.4)	<.001
Sometimes	512 (22.9)	191 (19.3)	
Often	298 (13.3)	400 (40.3)	
Have breaks when learning			
Occasion	824 (36.8)	362 (36.5)	.003
Sometimes	1092 (48.8)	441 (44.5)	
Often	323 (14.4)	188 (19.0)	
Remind of taking breaks from parents			
Occasion	100 (4.5)	51 (5.1)	.168
Sometimes	691 (30.9)	333 (33.6)	
Often	1448 (64.7)	607 (61.2)	
Education of father			
Junior and below	835 (37.3)	341 (34.4)	.13
Senior high school	534 (23.8)	224 (22.6)	
Junior College	346 (15.5)	154 (15.5)	
Bachelor’s degree	468 (20.9)	236 (23.8)	
Master’s degree	50 (2.2)	32 (3.2)	
Doctor’s degree	6 (0.3)	5 (0.5)	
Education of mother			
Junior and below	994 (44.4)	384 (38.7)	.002
Senior high school	463 (20.7)	215 (21.7)	
Junior College	384 (17.2)	172 (17.3)	
Bachelor’s degree	368 (16.4)	193 (19.5)	
Master’s degree	24 (1.1)	25 (2.5)	
Doctor’s degree	6 (0.3)	3 (0.3)	
Have siblings			
No	1359 (60.7)	486 (49.0)	<.001
Yes	880 (39.3)	506 (51.0)	

Means (±standard deviation) for continuous and percentage for classification variables.

* Compared between female and male groups.

### 3.2. The BMI and VI

The BMI categories in Table 2 compare the prevalence of the visually impaired (the right eye). The prevalence of severe vision impairment was 22.4%, 21.6%, 15.4%, and 21.4% in the underweight, normal, overweight, and obese participants, respectively. The prevalence of VI in each group has no significant difference (*P* value = .071).

**Table 2:** Physical status and vision impairing prevalence (n (%)).

	No vision impairment	Mild vision impairment	Moderate vision impairment	Severe vision impairment	*P* value*
Physical status type					
Underweight (n = 700)	110 (15.7)	82 (11.7)	351 (50.1)	157 (22.4)	.071
Normal (n = 1940)	341 (17.6)	239 (12.3)	940 (48.5)	420 (21.6)	
Overweight (n = 376)	75 (19.9)	38 (10.1)	205 (54.5)	58 (15.4)	
Obese (n = 215)	43 (20.0)	18 (8.4)	108 (50.2)	46 (21.4)	

VI was classified as mild (20/40 to 20/70), moderate (20/70 to 20/200), and severe (20/200 to 20/400) visual impairment. Physical status type was expressed by a BMI, which categorized into underweight (<18.5 kg/m^2^), normal weight (<24 kg/m^2^), overweight (<28 kg/m^2^), and obese (≥28 kg/m^2^). BMI = weight(kg)/square[weight(m)].

* Compared between VI and physical status.

### 3.3. Bivariate logistic regression analysis of factors of VI

In the bivariate logistic analysis, BMI was not related to VI for both mild-severe VI and moderate-severe VI. Height and weight were negatively related to VI. Besides the body size, other factors, such as being female, father or mother having myopia, late owning a smartphone, occasional or disliked exercise, few breaks when learning, and a low education level of the father or mother, were significantly related to the presence of VI. See Table 3.

**Table 3:** Results of the bivariate logistics regression analysis.

	Vision impairment 1 (mild-severe)			Vision impairment 2 (moderate-severe)		
	Right eye < 20/40			Right eye < 20/70		
	Odds ratio	95% confidence interval	*P* value*	Odds ratio	95% confidence interval	*P* Value†
BMI (from underweight to obesity)	0.986	0.967–1.005	.154	0.996	0.979–1.013	.639
Height	0.969	0.958–0.979	<.001	0.975	0.966–0.984	<.001
Weight	0.990	0.984–0.995	<.001	0.993	0.988–0.998	.007
Gender (male vs female)	0.619	0.513–0.747	<.001	0.712	0.606–0.836	<.001
Father have VI (vs normal)	1.633	1.324–2.014	<.001	1.528	1.289–1.811	<.001
Mother have VI (vs normal)	1.529	1.242–1.883	<.001	1.297	1.097–1.533	.002
Time of owning smartphone (from primary school to now)	1.158	1.050–1.278	.003	1.146	1.056–1.243	.001
Boarding from middle school (vs no boarding)	0.775	0.624–0.963	.021	1.165	0.956–1.419	.129
Boarding from high school (vs no boarding)	0.840	0.673–1.048	.122	1.362	1.136–1.632	.001
Living caregivers (parents vs others)	0.913	0.717–1.164	.465	0.976	0.800–1.192	.815
Screens spending the most time (phone vs others)	0.926	0.656–1.306	.661	0.898	0.674–1.198	.178
Doing exercise (from occasion to often)	0.722	0.629–0.828	<.001	0.751	0.671–0.841	<.001
Like doing exercise (from dislike to like)	0.720	0.647–0.802	<.001	0.760	0.693–0.832	<.001
Breaks when learning (from occasion to often)	0.649	0.570–0.739	>.001	0.676	0.606–0.755	<.001
Reminder of break of using eye from parents (from occasion to often)	1.088	0.932–1.268	.285	1.044	0.915–1.189	.520
Education of father (from low to high)	0.906	0.884–0.973	.006	0.893	0.841–0.949	<.001
Education of mother (from low to high)	0.833	0.774–0.896	>.001	0.844	0.793–0.898	<.001
No siblings (yes vs no)	0.894	0.745–1.073	.228	0.843	0.723–0.981	.028

* Compared between mild vision impairment and each risk factor.

† Compared between moderate vision impairment and each risk factor.

### 3.4. Multiple logistic regression analysis of body size and VI

In the multiple logistic analysis, after adjusting all available confounders, physical status, including height, weight, and BMI, had no associations with the risk VI (from mild to severe) for both sexes. For females, the ORs were 1.002 (95% CI = 0.974–1.032, *P* = .877), 0.998(95% CI = 0.967–1.010, *P* = .298), and 0.999(95% CI = 0.989–1.009, *P* = .860) for BMI, height, and weight, respectively. For males, the ORs were 0.988 (95% CI = 0.955–1.021, *P* = .459), 0.980 (95% CI = 0.954–1.006, *P* = .135), and 0.995 (95% CI = 0.985–1.004, *P* = .285) for BMI, height, and weight, respectively. See Table 4.

**Table 4:** Results of the multiple logistics regression analysis between VI and body size.

	Vision impairment (Right eye, visual acuity < 20/40)					
	Female			Male		
	Odds ratio*	95% confidence interval	*P* value	Odds ratio*	95% confidence interval	*P* value
BMI (from underweight to obesity)	1.002	0.974–1.032	.877	0.988	0.955–1.021	.459
Height	0.998	0.967–1.010	.298	0.980	0.954–1.006	.135
Weight	0.999	0.989–1.009	.860	0.995	0.985–1.004	.285

* Adjusted by father/mother education and myopia, boarding status in middles school, caregiver, screen type and time, exercise, reminders and breaks for eye rest, siblings.

## 4. Discussion

Our study found no relationship between body size and VI prevalence in first-year university students. These results were consistent with some previous research. A study reported^[14]^ that is no association among height, weight, or BMI with myopia in 19-year-old males. Furthermore, an Israeli study on participants aged 17 to 19 reported that myopia is not related to body stature,^[15]^ and another study showed no significant relation between height and myopia.^[16]^ However, many reports showed that body stature was related to myopia or VI prevalence, especially in younger students.^[17,18]^

In the process of human development from infants to adults, refractive status is determined by balancing the refractive power of the cornea and lens with the AL of the eyes.^[19,20]^ A human was born with physiologic hyperopia (+2.50 to +3.00 D),^[21,22]^ called hyperopia reserve. As the body grows, human hyperopia refractions go towards emmetropia. This process is called emmetropization and is usually finished at 15 years old^[23]^ with the emmetropia refractive status (−0.50 to +0.50 D).^[24]^ Ocular dimension change was concomitant with physical development in children.^[25]^ Therefore, myopia may occur due to growth spurts in childhood because of prolonged AL.^[26,27]^ Height being positively associated with AL has been confirmed in many studies.^[10,17,28]^ It can be explained in previous reports why height and VI prevalence were significantly related in younger children. However, when body development stops at age 16 to 18, near-sight work and overusing eyes can keep AL elongation going through the regulation of extraocular and ciliary muscles, which can continue to attenuate the influence of physical development on VI. Our study confirmed our hypothesis that physical status might not be related to VI in young adults.

Subjects in our study have high academic performance, which requires long-time, near-sight work. Previous studies have found a definite correlation between higher educational levels and VI.^[9,29]^ Also, a high prevalence of myopia was found in urban areas than in rural regions,^[9]^ which indicated that near work and less time outdoors are the major risk factors for VI. Therefore, it is less likely for us to observe the influence of physical status on VI in first-year students at a prestigious university.

Interestingly, reminders for the eye-using break are likely protective factors. However, in our multiple logistic regression, it was negatively related to VI. It might be the reverse causality because severe VI may draw parents more attention to remind their children.

### 4.1. Strengthening and limitations

One of the strengths of this study was the high homogeneity of the subjects, which excludes many confounding factors such as age, education level, and academic performance that may exist in other studies. Furthermore, we investigated the relationships between physical and mild and moderate VI, respectively, which confirmed our results in different conditions. Besides, we adjusted the relationship with various lifestyle factors, making our results reliable.

Unfortunately, we did not measure AL at this time; that was a limitation of our study, so we cannot confirm if there is a relationship between AL and body size, which can explain the relationship between VI and body stature. Only one university was included in this study, which may not be generalized to those of the same age. Furthermore, some other confounding factors, such as residence place, sleep duration, studying hours, leisure activities, and myopic correction, were not collected and analyzed, which can bias our results. Despite these limitations, our reports provide valuable references regarding the relationship between VI and body status in young adults.

## 5. Conclusion

In conclusion, the relationship between body size and VI confirmed in children disappeared in young adults with high academic achievements. Our study found no relationship between body size, such as weight, height, and BMI, and VI prevalence in first-year university students. The cessation of physical growth, combined with potential eye strain resulting from overuse, may mitigate any previously observed positive associations between physical status and VI in children. However, the results showed activities like doing outdoor exercise or having breaks for using the eyes when learning are protective factors against VI, and encouraging these protective activities will be beneficial in schools or in communities. More studies are warranted to further confirm the relationship between body size and VI in young adults.

## Author contributions

**Conceptualization:** Xiaoya Qi.

**Data curation:** Jing Zhang.

**Formal analysis:** Jing Zhang.

**Methodology:** Xiaoya Qi.

**Writing – original draft:** Jing Zhang, Ruili Li, Yong Zhang, Cuihong Li, Bingwu Xu, Xiaoya Qi.

**Writing – review & editing:** Jing Zhang, Ruili Li, Yong Zhang, Cuihong Li, Bingwu Xu, Xiaoya Qi.

## References

[1] Holden BA Fricke TR Wilson DA. Global prevalence of myopia and high myopia and temporal trends from 2000 through 2050. Ophthalmology. 2016;123:1036–42. 26875007 10.1016/j.ophtha.2016.01.006

[2] Chen J He X Wang J. [Forcasting the prevalence of myopia among students aged 6-18 years in China from 2021 to 2030.] Zhonghua Yan Ke Za Zhi. 2021;57:261–7. 33832050 10.3760/cma.j.cn112142-20201228-000851

[3] McCarty CA Taylor HR. Myopia and vision 2020. Am J Ophthalmol. 2000;129:525–7. 10764864 10.1016/s0002-9394(99)00444-4

[4] Wang M Cui J Shan G. Prevalence and risk factors of refractive error: a cross-sectional Study in Han and Yi adults in Yunnan, China. BMC Ophthalmol. 2019;19:1–12. 30683073 10.1186/s12886-019-1042-0 PMC6347814

[5] Wang X He H Wang X. Prevalence and risk factors of myopia in Han and Yugur older adults in Gansu, China: a cross-sectional study. Sci Rep. 2020;10:8249. 32427926 10.1038/s41598-020-65078-x PMC7237487

[6] Yang F Yang C Liu Y. Associations between body mass index and visual impairment of school students in central China. Int J Environ Res Public Health. 2016;13:1024. 27763567 10.3390/ijerph13101024 PMC5086763

[7] Wong TY Foster PJ Johnson GJ. The relationship between ocular dimensions and refraction with adult stature: the Tanjong Pagar Survey. Invest Ophthalmol Vis Sci. 2001;42:1237–42. 11328733

[8] Saw S-M Carkeet A Chia K-S. Component dependent risk factors for ocular parameters in Singapore Chinese children. Ophthalmology. 2002;109:2065–71. 12414416 10.1016/s0161-6420(02)01220-4

[9] Rim TH Kim SH Lim KH. Body stature as an age-dependent risk factor for Myopia in a South Korean Population. Semin Ophthalmol. 2017;32:326–36. 27058304 10.3109/08820538.2015.1088554

[10] Saw S-M Chua W-H Hong C-Y. Height and its relationship to refraction and biometry parameters in Singapore Chinese children. Invest Ophthalmol Vis Sci. 2002;43:1408–13. 11980854

[11] Organization WH. International Statistical Classification of Diseases and Related Health Problems: Alphabetical Index. World Health Organization; 2004.

[12] Sun J Zhou J Zhao P. High prevalence of myopia and high myopia in 5060 Chinese university students in Shanghai. Invest Ophthalmol Vis Sci. 2012;53:7504–9. 23060137 10.1167/iovs.11-8343

[13] Chen C Lu FC; Department of Disease Control Ministry of Health, PR China. The guidelines for prevention and control of overweight and obesity in Chinese adults. Biomed Environ Sci. 2004;17(Suppl):1–36. 15807475

[14] Jung S-K Lee JH Kakizaki H. Prevalence of myopia and its association with body stature and educational level in 19-year-old male conscripts in Seoul, South Korea. Invest Ophthalmol Vis Sci. 2012;53:5579–83. 22836765 10.1167/iovs.12-10106

[15] Rosner M Laor A Belkin M. Myopia and stature: findings in a population of 106,926 males. Eur J Ophthalmol. 1995;5:1–6. 7795395 10.1177/112067219500500101

[16] Saw S-M Chan Y-H Wong W-L. Prevalence and risk factors for refractive errors in the Singapore Malay Eye Survey. Ophthalmology. 2008;115:1713–9. 18486221 10.1016/j.ophtha.2008.03.016

[17] Zhang J Hur Y-M Huang W. Shared genetic determinants of axial length and height in children: the Guangzhou twin eye study. Arch Ophthalmol. 2011;129:63–8. 21220630 10.1001/archophthalmol.2010.323

[18] Peng R Li S Zhang H. Weight status is associated with blood pressure, vital capacity, dental decay, and visual acuity among school-age children in Chengdu, China. Ann Nutr Metab. 2017;69:237–45. 10.1159/000454888 28013297

[19] Goss DA Cox VD Herrin-Lawson GA. Refractive error, axial length, and height as a function of age in young myopes. Optom Vis Sci. 1990;67:332–8. 2367086 10.1097/00006324-199005000-00006

[20] Morgan IG Ohno-Matsui K Saw S-M. Myopia. Lancet. 2012;379:1739–48. 22559900 10.1016/S0140-6736(12)60272-4

[21] Axer-Siegel R Herscovici Z Davidson S. Early structural status of the eyes of healthy term neonates conceived by in vitro fertilization or conceived naturally. Invest Ophthalmol Vis Sci. 2007;48:5454–8. 18055792 10.1167/iovs.07-0929

[22] Rozema JJ Herscovici Z Snir M. Analysing the ocular biometry of new-born infants. Ophthalmic Physiol Opt. 2018;38:119–28. 29285779 10.1111/opo.12433

[23] Limony Y Kozieł S Friger M. Age of onset of a normally timed pubertal growth spurt affects the final height of children. Pediatr Res. 2015;78:351–5. 26020145 10.1038/pr.2015.104

[24] Morgan IG Rose KA Ellwein LB; Refractive Error Study in Children Survey Group. Is emmetropia the natural endpoint for human refractive development? An analysis of population-based data from the refractive error study in children (RESC). Acta Ophthalmol. 2010;88:877–84. 19958289 10.1111/j.1755-3768.2009.01800.x PMC2891782

[25] Prashar A Hocking PM Erichsen JT. Common determinants of body size and eye size in chickens from an advanced intercross line. Exp Eye Res. 2009;89:42–8. 19249299 10.1016/j.exer.2009.02.008

[26] Baird PN Schäche M Dirani M. The GEnes in Myopia (GEM) study in understanding the aetiology of refractive errors. Prog Retin Eye Res. 2010;29:520–42. 20576483 10.1016/j.preteyeres.2010.05.004

[27] Zheng Y Lavanya R Wu R. Prevalence and causes of visual impairment and blindness in an urban Indian population: the Singapore Indian Eye Study. Ophthalmology. 2011;118:1798–804. 21621261 10.1016/j.ophtha.2011.02.014

[28] Xiang F He M Zeng Y. Increases in the prevalence of reduced visual acuity and myopia in Chinese children in Guangzhou over the past 20 years. Eye. 2013;27:1353–8. 24008929 10.1038/eye.2013.194 PMC3869515

[29] Goldschmidt E Jacobsen N. Genetic and environmental effects on myopia development and progression. Eye (Lond). 2014;28:126–33. 24357837 10.1038/eye.2013.254 PMC3930266

